# Comparative Study of In Situ Techniques to Enlarge Gold Nanoparticles for Highly Sensitive Lateral Flow Immunoassay of SARS-CoV-2

**DOI:** 10.3390/bios11070229

**Published:** 2021-07-08

**Authors:** Vasily G. Panferov, Nadezhda A. Byzova, Sergey F. Biketov, Anatoly V. Zherdev, Boris B. Dzantiev

**Affiliations:** 1 Research Center of Biotechnology of the Russian Academy of Sciences, A.N. Bach Institute of Biochemistry, 119071 Moscow, Russia; panferov-vg@mail.ru (V.G.P.); nbyzova@inbi.ras.ru (N.A.B.); zherdev@inbi.ras.ru (A.V.Z.); 2 State Research Center for Applied Microbiology & Biotechnology, 142279 Obolensk, Moscow Region, Russia; biketov@obolensk.org

**Keywords:** immunochromatography, point-of-care testing, limit of detection, antigen test, coronavirus, receptor-binding domain, silver enhancement, gold enhancement, galvanic replacement, spike protein

## Abstract

Three techniques were compared for lowering the limit of detection (LOD) of the lateral flow immunoassay (LFIA) of the receptor-binding domain of severe acute respiratory syndrome-related coronavirus 2 (SARS-CoV-2) based on the post-assay in situ enlargement of Au nanoparticles (Au NPs) on a test strip. Silver enhancement (growth of a silver layer over Au NPs—Au@Ag NPs) and gold enhancement (growth of a gold layer over Au NPs) techniques and the novel technique of galvanic replacement of Ag by Au in Au@Ag NPs causing the formation of Au@Ag-Au NPs were performed. All the enhancements were performed on-site after completion of the conventional LFIA and maintained equipment-free assay. The assays demonstrated lowering of LODs in the following rows: 488 pg/mL (conventional LFIA with Au NPs), 61 pg/mL (silver enhancement), 8 pg/mL (galvanic replacement), and 1 pg/mL (gold enhancement). Using gold enhancement as the optimal technique, the maximal dilution of inactivated SARS-CoV-2-containing samples increased 500 times. The developed LFIA provided highly sensitive and rapid (8 min) point-of-need testing.

## 1. Introduction

Lateral flow immunoassay (LFIA) is the analytical method that combines the interaction of antibodies with antigens and affinity separation of the formed complexes during their migration along porous membranes [1,2]. Because of their simplicity, rapidity, and low cost, LFIA tests are used widely for point-of-need detection of pathogens and a wide range of xenobiotic compounds [3,4].

However, the main limitation of LFIA is its high limit of detection (LOD), which restricts its application for analyzing low-abundance targets. Many approaches to reduce the LOD of LFIA have been developed [5]. Focusing on equipment-free colorimetric LFIA as the most convenient for practical use, the assortment is limited to two major approaches [6]: (a) changes in the physicochemical methods of the colorimetric label (i.e., the use of the nanosized labels that can be registered in lower concentrations [7]) and (b) increase in the amount of the label/derivative products (e.g., aggregation of nanoparticles [8], releasing of registered molecules from nanocarriers [9], and catalytic generation of registered molecules [10]).

Gold nanoparticles (Au NPs) are one of the most widely used markers in LFIA because of their simple and reproducible synthesis and bioconjugation as well as their plasmonic characteristics and tunable optical properties [11]. The size and shape of Au NPs influence the optical properties, and in the first view, the use of larger Au NPs is beneficial for highly sensitive detection. Khlebtsov et al. [12] studied minimal content of Au NPs in lateral flow membranes causing visually detected coloration. They showed that larger Au NPs were detected at the lower amounts—6.5 × 10^7^ and 1.4 × 10^5^ nanoparticles per mm^2^ of the membrane with pore size 10 µM (conventionally used nitrocellulose membrane for LFIA) for Au NPs with the diameters 16 and 115 nm, respectively. Although the use of larger Au NPs is desirable, in practice, the use of large Au NPs is limited by poor migration through porous membranes.

The use of nonspherical Au NPs was reported for LOD reduction. Serebrenikova et al. [13] reported five-times lower LOD for the LFIA of procalcitonin achieved by the replacement of spherical Au NPs with hierarchical Au NPs. Zhang et al. [14] reported the effect of different nonspherical Au NPs on LOD and achieved 100-times lower LOD using tipper flower-like Au NPs. The reduction in LOD achieved by the use of nonspherical Au NPs could be explained by the higher molar extinction coefficient of the particles (as well as for larger spherical Au NPs); thus, such Au NPs provide higher coloration intensity with the same number of particles and facilitate the visual registration of the colored test, which is not detectable for conventional spherical Au NPs.

To overcome the limitations of the initial size of Au NPs, various methods for post-assay modifications (including enlargement [15], change of shape [16], and chemical composition—the overgrowth of Ag [17], Pt [18], and Cu [19]) have been reported. Among them, the deposition of a silver layer over Au NP seeds (silver enhancement) is a common method for LOD reduction, which is widely used in LFIA and other techniques [20]. The growth of the coloration after post-assay enhancement is explained by the higher molar extinction coefficient of the larger and hierarchically shaped nanoparticles [21].

In this article, we report (a) the new approach of LFIA enhancement combining silver enhancement and galvanic replacement of Ag with [AuCl_4_]^−^ ions (called galvanic-assisted Au deposition) and (b) the comparison of three enhancement methods of LFIA with Au NPs—silver enhancement, galvanic-assisted Au deposition, and gold enhancement. Although the silver and gold enhancement methods are well known in the literature, the comparison of these approaches for the in situ enhancement of LFIA has not been reported.

As the target analyte, we selected the receptor-binding domain (RBD) of the spike protein of severe acute respiratory syndrome-related coronavirus 2 (SARS-CoV-2). The accessibility of rapid, inexpensive, and reliable antigen testing is one of the keys (along with PCR and serodiagnostics) to controlling SARS-CoV-2 infection [22]. Highly sensitive diagnostics are especially necessary because the SARS-CoV-2 content in throat swab samples varies widely (from 641 to 1.34 × 10^11^ copies of N-gene) [23], and the ability to detect its low concentrations will improve the efficiency of diagnostics. Although the majority of LFIAs that are focused on are serodiagnostic (i.e., detect IgG and IgM) [22], the demand for LFIAs of the SARS-CoV-2 antigen is rapidly growing (there are more than 100 commercially available/in-development tests as of May 2021). Most of these tests utilize Au NPs as a colorimetric label; thus, reported enhancement strategies can be applied for them. We believe that the results presented in this work will contribute to the area of highly sensitive point-of-care SARS-CoV-2 diagnostics as well as diagnostics for other clinically important pathogens.

## 2. Materials and Methods

### 2.1. Materials

The pair of monoclonal antibodies (mAb) to RBD (clones 5324 and 5308) and recombinant RBD were provided by Hy Test (Moscow, Russia). SARS-CoV-2-inactivated virions (2019-nCoV/Victoria/1/2020) were provided by State Research Center of Virology and Biotechnology VECTOR (Novosibirsk area, Russia). Recombinant staphylococcal protein A was purchased from Imtek (Russia, Moscow). Saliva samples were obtained from a healthy volunteer after obtaining written and informed consent. Tetrachloroauric (III) acid (H[AuCl_4_]), silver nitrate, hydroquinone, hydrogen peroxide (30%), and bovine serum albumin (BSA) were purchased from Sigma Aldrich (St. Louis, MO, USA). All salts and acids were from Khimmed (Moscow, Russia). Deionized water produced by Milli-Q (Billerica, MA, USA) was used for the preparation of buffers.

Nitrocellulose membranes (CNPC-15), membrane for conjugate storage (PR-R5), a membrane for applying the sample (GFB-R4), and the final adsorbing membrane (AP045) were purchased Advanced Microdevices (Haryana, India).

### 2.2. Synthesis of Au NPs

Synthesis of Au NPs was performed following modified procedure of H[AuCl_4_] reduction by citrate [24]. An aliquot of H[AuCl_4_] (97 mL, 0.01%) was heated to 100 °C, and sodium citrate (3 mL, 1%) was added. The solution was kept at 100 °C for 30 min and stirred vigorously.

### 2.3. Synthesis of Au NP–mAb Conjugates

Conjugates were synthesized by physical adsorption of mAb on Au NPs. An aliquot of Au NP (1 mL, A_520_ = 1, pH = 8.5) was mixed with mAb (clone 5324, final concentration was equal to 12 µg) at room temperature for 2 h. After this step, BSA was added (0.25%). Conjugates were separated at 18,000× *g* for 25 min, and conjugates were resuspended in 20 mM Tris with 0.25% BSA, Tween-20, 1% sucrose, and 0.05% sodium azide.

### 2.4. Characteristics of Nanoparticles

The size and shape of nanoparticles were studied using the Jeol JEM-1400 transmission electron microscope (Tokyo, Japan) combined with the energy-dispersive spectrometer Oxford Instruments INCA Energy TEM 350 (High Wycombe, UK). The size and morphology of nanoparticles into membranes were studied using a scanning electron microscope Tescan MIRA 3 LMU (Brno, Czech Republic). Membranes were coated with gold using Quorum Technologies Q150T ES coater (Laughton, UK).

### 2.5. Preparation of Test Strips

Protein A (0.5 mg/mL) and mAb (clone 5308, 1 mg/mL) were dispensed on control zone (CZ) and test zone (TZ), respectively, of the CNPC-15 membrane by an Image Technology IsoFlow dispenser (Hanover, USA) at 0.15 µL/mm. The Au NP–mAb conjugate (A_520_ = 4) was applied to the PR-R5 membrane (1.5 µL/mm). All the membranes were dried at room temperature for 12 h; afterward, GFB-R4 and AP045 were glued. The assembled membranes were cut into test strips (3 mm wide) using A-Point Technologies Index Cutter-1 (Gibbstown, NJ, USA). The test strips were stored at room temperature in zipper bags.

### 2.6. Performance of Conventional and Enhanced LFIA

LFIA was performed using artificially contaminated saliva with RBD and SARS-CoV-2-inactivated virions. Saliva was dissolved five times with 50 mM potassium phosphate buffer, pH 7.4 NaCl was added to the final concentration at 200 mM, and Triton X-100 was added at 0.5%. Saliva was used for the dilution of the samples. The test strips were placed into analyzed probe sample (100 µL). The conventional LFIA coloration of TZs and CZs was detected after 5 min.

For silver enhancement, the test strips after conventional LFIA were used. The enhancement solution was prepared by mixing equal volumes of 2% hydroquinone in 10 mM citric buffer, pH = 4.0 and 0.2% silver nitrate in water. The test strips were washed with distilled water, and the enhancement solution (10 µL) was added and incubated for 2 min.

For galvanic-assisted Au deposition, the test strips after silver enhancement were used. Afterward, 10 µL of H[AuCl_4_] (5 mM) was added and incubated for 1 min.

For gold enhancement, the test strips after conventional LFIA were used. Equal volumes of 5 mM H[AuCl_4_] and 1 M H_2_O_2_ were mixed, and the obtained enhancing solution (10 µL) was added and incubated for 3 min.

After completion of the assay, the test strips were scanned using a conventional office scanner (Canon 9000F Mark II). The grayscale digital images were analyzed using TotalLab TL 120 (Nonlinear Dynamics, UK). The calibration plots (mean colorimetric signals of TZs against RBD concentration/virion dilution) were obtained using Origin 9.5 software (Origin Lab, Northampton, MA, USA). The LOD values of conventional and enhanced LFIA were calculated as the RBD concentration or dilution of SARS-CoV-2-containing samples corresponding to a colorimetric signal higher than a mean (*n* = 5) colorimetric signal for negative probe (blank probe without RBD, A_blank_) plus three standard deviations (SD) of A_blank_ − A_blank_ + 3SD.

## 3. Results and Discussion

### 3.1. Characteristics of Nanoparticles

**Figure 1:**
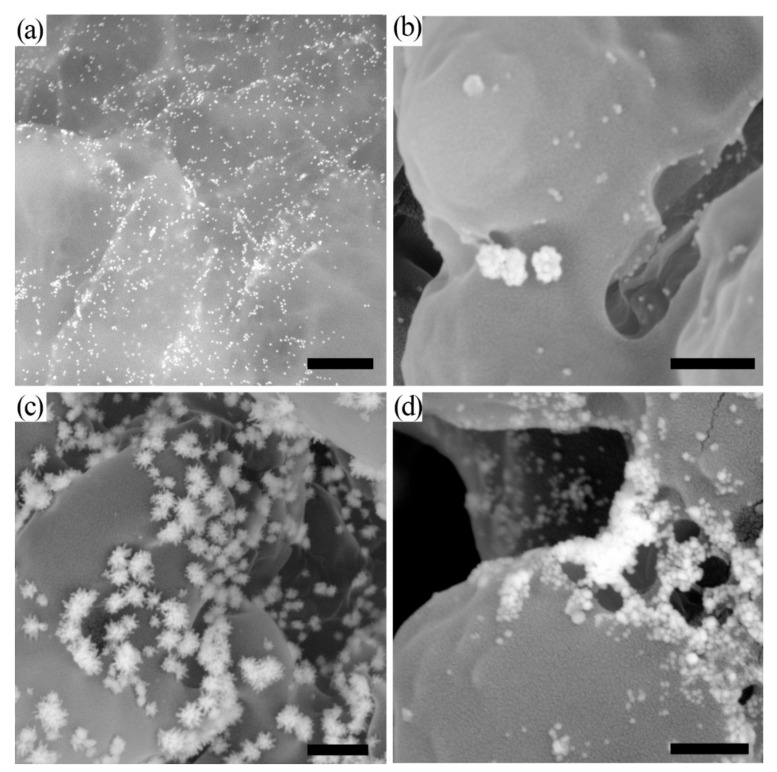
Microphotographs of nanoparticles in nitrocellulose membranes. (**a**) Au NPs; (**b**) Au@Ag NPs after silver enhancement; (**c**) Au@Ag-Au NPs after galvanic-assisted Au deposition; (**d**) Au NPs after gold enhancement. The bare scale is equal to 500 nm. Microphotographs were obtained using SEM operating in back-scattered electron detection mode.

In accordance with TEM results, the average diameter of the obtained Au NPs was equal to 22.8 ± 1.1 nm; the elongation coefficient (ratio of major axis length to the minor axis length) was equal to 1.07 ± 0.07. Au NPs were monodispered and non-aggregated (Figure S1). Thus, the synthesized Au NPs were applicable for conjugation with mAb and use in LFIA. The initial Au NPs (Figure 1a) acting as seeds for in situ enhancement were visualized by SEM on the membrane and demonstrated the expected shape and size.

All three performed enhancements led to the growth of NPs’ sizes (up to hundreds of nm). Because of the limited diffusion in a porous membrane, we observed both enlarged and relatively small nanoparticles (20–50 nm). The developed approaches include the step of silver/gold salt reduction catalyzed by Au NPs. Although the reduction initially occurs exclusively on seed Au NPs, the prolonged incubation of enhancement solution leads to the reduction of metal salts and the formation of new seed nanoparticles [25]. This phenomenon leads to nonspecific coloration and can be avoided by washing the precursors or selecting the proper time for measurements. To avoid out-of-seed reduction of metal salts during SEM, the test strips were washed with water.

After silver enhancement, large nonspherical particles were observed (Figure 1b), indicating the reduction of silver salt over the Au seeds and the formation of Au@Ag core@shell nanoparticles. Besides the enlarged Au NPs, initial Au NPs were observed as well. EDS analysis confirmed the presence of Ag and Au in the formed nanoparticles (Figure S2a). EDS mapping confirmed the formation of the silver shell around Au NPs acting as the cores (Figure S4). Previously published results confirmed the complete coverage of the Au core with a silver layer [26,27]. For Au@Ag nanoparticles with a thin Ag layer, electron transfer from Au core atoms to the Ag shell, stabilizing the silver layer to oxidation, was reported in [26].

Newly developed galvanic-assisted Au deposition led to the formation of hierarchically shaped nanoparticles (Figure 1c). The novelty of the reported approach is related to the post-assay formation of nanoparticles directly on a test strip. In previous studies, authors synthesized nanoparticles in colloidal solution (overgrowth of Ag over Au NPs and consequent galvanic replacement) and used them for conjugation [28]. Although this approach facilitates the synthesis of core@shell nanoparticles, initial size restrictions remain unsolved. Using the post-assay enhancement strategy on the membrane overcomes the size limitations. Moreover, in comparison with nanoparticles obtained in colloidal solution (Figure S3), the nanoparticles formed on the membrane had a significantly different morphology. TEM images (Figures S3 and S5a) demonstrate the formation of voids related to the galvanic replacement of silver atoms from the shell. EDS mapping confirmed the introduction of Au atoms in the shell and the presence of Cl in the nanoparticles. The mechanism of the chemical reaction of Ag with [AuCl_4_]^−^ was considered in several works, and the conjunction of galvanic replacement with the nanoscale Kirkendall effect was confirmed [29,30]. The observed spiky-shaped form of nanoparticles formed on the membrane (Figure 1c) may be explained by the precipitation of AgCl on the Au@Ag-Au NPs. The formations of Ag^+^ and Cl^−^ ions occur during the galvanic reaction Au@Ag_x_ + [AuCl_4_]^−^ → Au@Ag_(x−3y)_-Au_y_ + 3AgCl, where the deposition of single Au atoms leads to the replacement of three Ag atoms. The diffusion of Ag^+^ and Cl^−^ ions is limited in porous membranes, and the precipitate of low-soluble AgCl forms around nanoparticles [31]. Moreover, in porous membranes, the number of seed nanoparticles is not determined, and the reactivity of seeds is limited by blocking the surface of nanoparticles with membranes and proteins. Conversely, in a colloidal solution, the galvanic replacement is limited only by the diffusion of ions, and the reactivity of the particles is not limited by the shielding. The replacement of three Ag atoms and the deposition of only one Au atom led to the formation of voids (Figure S3). Galvanic-assisted Au deposition results in an increase in the Au concentration and a decrease in the Ag concentration in comparison with Au@Ag NPs (Figure S2b), which proves the deposition of Au and replacement of Ag. We hypothesized that the observed deposition of precipitate around nanoparticles (Figure 1c) may be beneficial because of the additional increase in the nanoparticles’ sizes.

Gold enhancement resulted in the formation of heterogeneous Au NPs (Figure 1d). Because of the limited diffusion, we observed both relatively small Au NPs (20–50 nm) with close-to-spherical shape and large (50–500 nm) nonspherical Au NPs. Compared to silver enhancement, gold enhancement did not require additional washing to deplete chloride and phosphate ions.

As shown in previous studies [32,33], the size and morphology of nanoparticles formed after enlargement on porous membranes significantly differs from the enlargement in homogenous conditions (i.e., in colloidal solution). The blockade of the nanoparticles’ surface by the membrane and adsorbed macromolecules leads to the limited diffusion of the enhancing reactants and uneven growth of nanoparticles in the various axes. Such conditions lead to the formation of large, nonspherical particles with a broad size distribution. The approach used in this paper of the post-assay in situ enlargement of nanoparticles facilitates the benefits of large nonspherical nanoparticles as the optical labels and avoids the limitations of poor stability and migration through porous membranes.

### 3.2. Lateral Flow Immunoassay of Receptor-Binding Domain in Saliva

**Figure 2:**
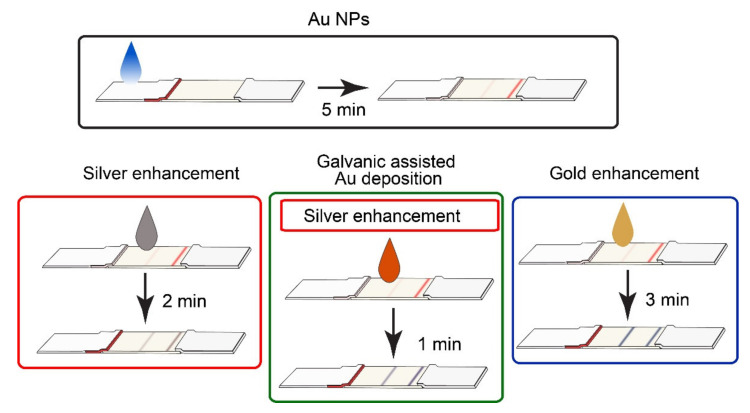
Four formats of LFIA used in the article. Test strips after completion of LFIA with Au NPs (black frame) were used for silver enhancement (red frame), galvanic-assisted Au deposition (green frame), and gold enhancement (blue frame). The galvanic-assisted Au deposition included silver enhancement (depicted as the nested red frame).

**Figure 3:**
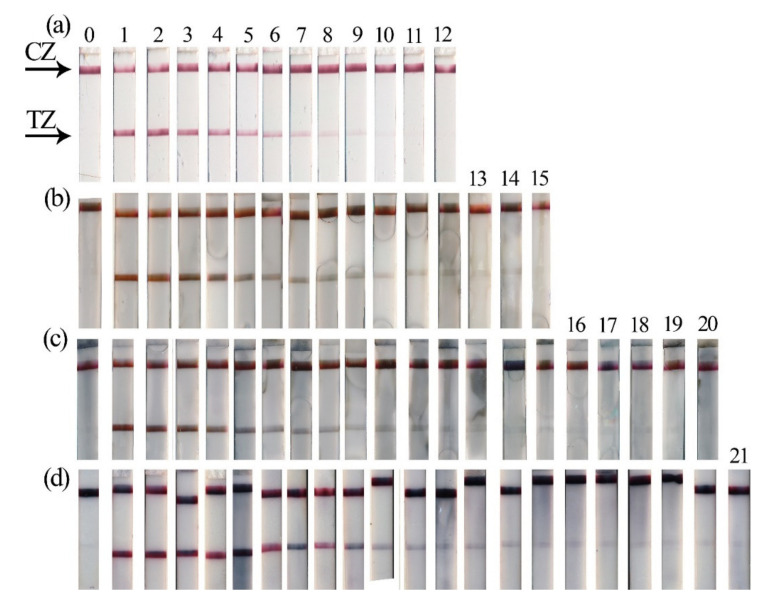

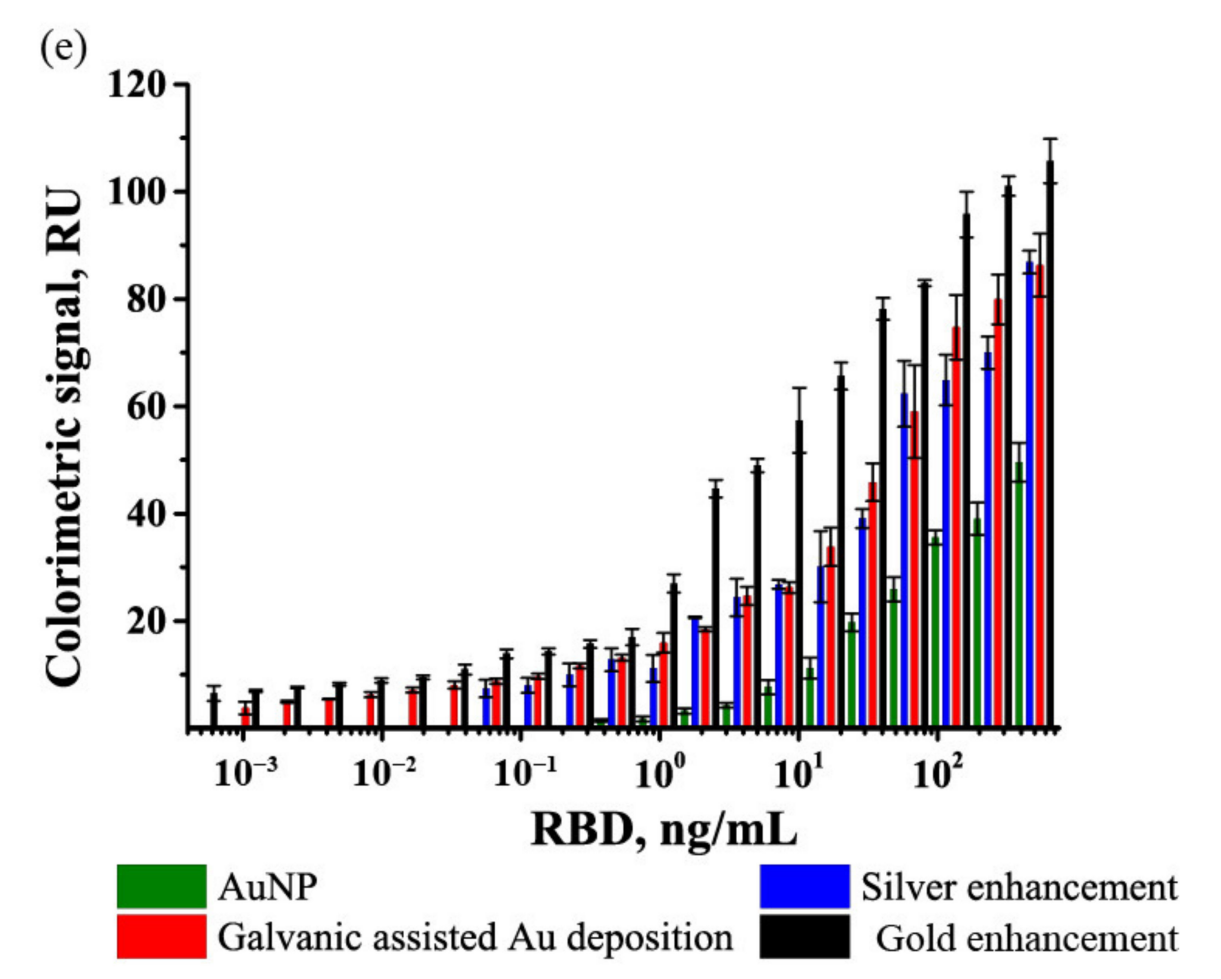
LFIA of RBD in artificially contaminated saliva. (**a**) Test strips with Au NPs; (**b**) Test strips after silver enhancement; (**c**) Test strips after galvanic-assisted Au deposition; (**d**) Test strips after gold enhancement: the numbers above the test strips show the concentration of RBD in the probe, in ng/mL, equal to 500 (1), 250 (2), 125 (3), 62.5 (4), 31.2 (5), 15.6 (6), 7.8 (7), 3.9 (8), 1.95 (9), 0.98 (10), 0.49 (11), 0.,24 (12), 0.12 (13), 0.06 (14), 0.03 (15). Concentration of RBD, in pg/mL, is equal to 15 (16), 7.5 (17), 3.8 (18), 1.9 (19), 0.9 (20), 0.05 (21), blank (0); (**e**) Dependences of the colorimetric signal in TZs versus RBD concentrations for various LFIA formats.

Four formats (one conventional format and three enhanced formats) of LFIA were performed (Figure 2). LFIA with Au NPs (Figure 2, Au NPs) was used as the conventional assay, and the benefits of the enhancement strategies (Figure 2, silver enhancement, galvanic-assisted Au deposition, gold enhancement) were determined by comparison of their LOD values (Figure 3e) and analytical features of the assays. All four formats were performed using the same antibodies and initial Au NPs; thus, we were able to determine the influence of the enhancement strategy (i.e., modification of nanoparticles in TZ) on LOD.

All formats were operated using the same principle of sandwich immunoassay. RBD was captured by mAb immobilized on Au NP and TZ (i.e., RBD was sandwiched between two mAb) during the migration of the liquid sample. The presence of RBD in saliva resulted in the formation of colored TZ. The coloration of CZ demonstrated the reliability of the assay. For the conventional LFIA with Au NPs, the LOD of RBD was equal to 488 pg/mL. Nonspecific coloration of TZ was not observed (Figure 3a).

After the completion of immunochemical reactions, three enhancement strategies were realized. Incubation times of enhancement solutions were empirically selected considering the minimal background staining.

For silver enhancement, two solutions containing AgNO_3_ and hydroquinone were mixed and incubated on the test strip. Catalyzed by Au NPs, the reduction of silver salt led to the formation of Au@Ag nanoparticles possessing higher coloration (Figure 3b). For the LFIA with silver enhancement, the LOD of RBD was equal to 61 pg/mL, and nonspecific coloration of TZ was not observed.

For galvanic-assisted Au deposition, test strips after completion of silver enhancement were used. Adding H[AuCl_4_] initiated the reaction of galvanic replacement and led to the formation of Au@Ag-Au nanoparticles. Newly formed Au@Ag-Au nanoparticles possessed higher coloration (Figure 3c) and facilitated the reduction of LOD of RBD to 8 pg/mL. Nonspecific coloration of TZ was observed for this enhancement strategy (Figure S6). Despite the fact that the galvanic-assisted Au deposition proposed in this work provides a modest reduction in LOD (around 8-fold in comparison with silver enhancement), its simplicity and rapidity make this strategy an attractive additional step. Considering the high reduction potential of [AuCl_4_]^−^/Au (0.99 V vs. SHE [31]), the developed approach can be performed for galvanic-assisted Au deposition using various core@shell nanoparticles to enhance LFIA (i.e., Au@Cu [19], Au@Pt [34]).

For gold enhancement, two solutions containing H[AuCl_4_] and H_2_O_2_ were mixed and incubated on the test strip. Catalyzed by Au NPs, the reduction of gold salt led to the formation of enlarged Au NPs and the growth of TZs’ and CZs’ coloration (Figure 3d). Nonspecific coloration of TZ was observed for this enhancing approach. However, using an optical quantification instrument facilitated the detection of RBD with LOD equal to 1 pg/mL (Figure S7).

Whereas the majority of publications about SARS-CoV-2 point-of-care diagnostics are focused on the use of LFIA for serodiagnosis [22], there are few papers about antigen-detecting LFIA. Grant et al. [35] reported a dipstick assay (i.e., conjugate and sample are premixed prior to insertion of nitrocellulose membrane) for nucleocapsid protein. Using colored latex beads as the label, the authors reported LOD equal to 0.65–3.03 ng/mL (for different manufacturers of recombinant protein). Liu et al. [36] reported LFIA with chemiluminescence detection and Co-Fe@hemin nanoparticles as a catalytic label. The developed LFIA provided highly sensitive detection of S-RBD protein (down to 0.1 ng/mL) within 16 min. Hristov et al. [37] compared the performance of six antibodies conjugated to Au NPs and seven S proteins in LFIA and reported a 0.07 nM LOD value. Guo et al. [38] reported that LFIA with mesoporous silica encapsulated up-conversion nanoparticles. The reported LFIA with fluorescent detection used test strips with two TZs to simultaneously detect S- and N-proteins of SARS-CoV-2. The LODs of the developed LFIA were equal to 1.6 and 2.2 ng/mL for S- and N-proteins, respectively.

All formats of LFIA studied in this research demonstrated no false-positive results. The used monoclonal antibodies bind specifically to RBD SARS-CoV-2, and the manufacturer declares the absence of cross-reactivity for closely related MERS-CoV, seasonal coronaviruses, influenza A and B, human respiratory syncytial virus, and adenovirus [39].

The considered approaches differ in potential applicability as point-of-care diagnostic tools. The possibilities of some approaches are limited by the high number of stages (i.e., the use of galvanic-assisted Au deposition requires three post-assay stages) and low stability of the components of the enhancement solution in liquid form (i.e., hydroquinone and silver salts for silver enhancement should be freshly prepared). Gold enhancement not only provides the lowest LOD but also facilitates the rapid and conventional assay’s procedure and is implemented using stable precursors. The performed evaluation of precursors’ costs (see Supplementary Materials, Section S6) showed that the developed test systems are cost-effective, and the use of additional reagents does not significantly increase their price (Table S1). Thus, the reported four formats of LFIA may be used as the single-use point-of-care test in resource-limited conditions.

### 3.3. Validation of LFIA in Testing Samples with Inactivated SARS-CoV-2 Virions

Considering all the benefits of gold enhancement, this format was selected for further development. LFIA with gold enhancement was used for recovery studies of artificially contaminated saliva. Quantitative correlation between spiked and measured concentrations of RBD in saliva demonstrated a high correlation both for conventional and gold-enhanced LFIAs (R^2^ = 0.98, Figure S8).

**Figure 4:**
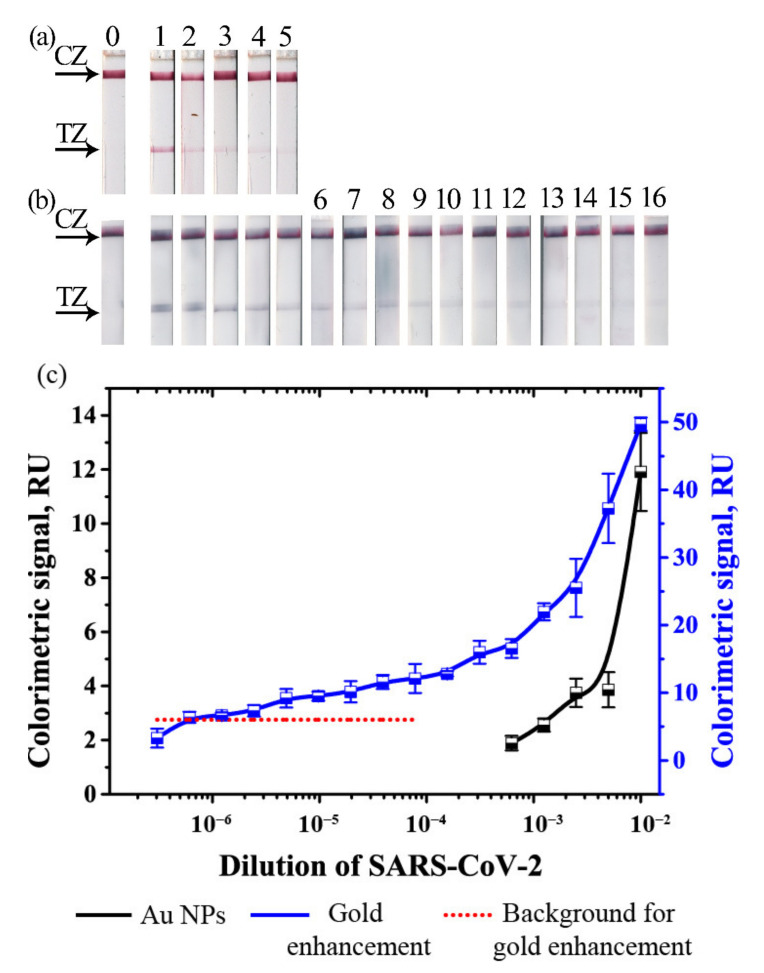
LFIA of SARS-CoV-2 virions in saliva. (**a**) Test strips with Au NPs; (**b**) Test strips after gold enhancement; (**c**) Calibration plots for LFIA with Au NPs and after gold enhancement.

Validation of LFIA was performed using inactivated SARS-CoV-2-containing samples. The virus was inactivated by treatment with β-propiolactone. Thus, the concentration of the epitopes could not be determined. Thus, instead of comparing concentrations of protein, we compared dilutions of the inactivated virus stock solution. The dilutions, which provided a detectable colorimetric signal, were compared for conventional LFIA and LFIA with gold enhancement (Figure 4).

Gold enhancement facilitated a significant increase in the colorimetric signals in the dilution range, where coloration of Au NPs was detected (10^−2^–10^−3^) and resulted in the formation of the visually detected coloration of TZs in the dilution range 10^−3^–10^−5^ (Figure 4a). Background staining limited the LOD of the LFIA in significantly higher dilutions in comparison with LFIA with Au NPs. The results (Figure 4b) showed that LFIA with gold enhancement detected significantly lower dilutions of SARS-CoV-2-containing samples (down to 819, 200 times, Figure 4b,c) in comparison with Au NPs (1600 times, Figure 3a,c).

The reported LFIA provides highly sensitive and rapid detection of SARS-CoV-2. Because of the simplicity of the assay, the reported LFIA can be used for self-testing in point-of-need conditions and as a complementary test to PCR. Such rapid and easy-to-use tests are in demand by public health [40]. LFIA antigen tests have already shown promising performance for mass population screening [41]. The expected further developments in the area of point-of-care detection of SARS-CoV-2 will be related to highly sensitive and multiplex assays. Multiplexing (i.e., detection with a single test SARS-CoV-2 and other respiratory viruses) will facilitate a more informative assay and distinguish COVID-19 and infections with similar symptoms [42]. Significant efforts have been made for the multiplexing of LFIA [43], and well-developed prototypes (i.e., LFIA with multiple test-zones [44]) can be easily created. The high sensitivity of LFIA tests is a mandatory requirement for their clinical use [45,46]. Highly sensitive multiplex LFIA is achieved by the use of nanoparticles that can be detected at low concentrations [47] or by applying enhancement approaches [6]. Such easy-to-use Au NP-based tests may facilitate highly sensitive and multiplex detection by spatial separation of test zones on the membrane [43], barcode strategy [48], or fluorescent encoding [49].

## 4. Conclusions

We developed a lateral flow immunoassay of the receptor-binding domain of SARS-CoV-2 using spherical Au NPs and compared three approaches for reducing the limit of detection. The strategies were based on post-assay in situ modification of Au nanoparticles and thus are applicable for the majority of lateral flow immunoassays. We found that gold enhancement provides the lowest limit of detection of receptor-binding domain—1 pg/mL in artificially contaminated saliva. The developed assay with gold enhancement was able to detect inactivated SARS-CoV-2 virions in 500-times lower dilutions in comparison to the conventional assay. This approach utilizes stable reagents, facilitates rapid (8 min) and easy point-of-need testing, and potentially could be used in nonlaboratory practice.

## Data Availability

Data are available upon reasonable request from corresponding author.

## Supplementary Materials

The following are available online at https://www.mdpi.com/article/10.3390/bios11070229/s1, Figure S1: Characteristics of Au NPs, Figure S2: EDS spectra of nanoparticles, Figure S3: Microphotographs of Au@Ag-Au nanoparticles, Figure S4: Microphotographs of Au@Ag NPs, Figure S5: Microphotographs of Au@Ag-Au NPs. Figure S6: Calibration curve for LFIA after galvanic-assisted Au deposition at low RBD concentrations, Figure S7: Calibration curve for LFIA after gold enhancement at low RBD concentrations, Figure S8: Correlation between added and detected concentrations of RBD in saliva. Table S1. Consumption of Au and Ag precursors for the developed LFIA formats.
