# Supplementary material for: Comparative Study of In Situ Techniques to Enlarge Gold Nanoparticles for Highly Sensitive Lateral Flow Immunoassay of SARS-CoV-2
Source: Biosensors (Basel). 2021 Jul 8;11(7):229. doi: 10.3390/bios11070229 (PMC8301938; doi:10.3390/bios11070229)
Supplement: Supplementary file 1 [file biosensors-11-00229-s001.zip › biosensors-1262806-supplementary.pdf]

Supplementary materials

Comparative study of in situ techniques to enlarge gold nanoparticles for highly sensitive lateral flow immunoassay of SARS-CoV-2

Vasily G. Panferov[1], Nadezhda A. Byzova[1], Sergey F. Biketov[2], Anatoly V. Zherdev[1], Boris B. Dzantiev[1]

1 – A.N. Bach Institute of Biochemistry, Research Center of Biotechnology of the Russian Academy of Sciences, 119071 Moscow, Russia

2 – State Research Center for Applied Microbiology & Biotechnology, 142279 Obolensk, Moscow region, Russia

S1. Transmission electron microscopy of Au nanoparticles

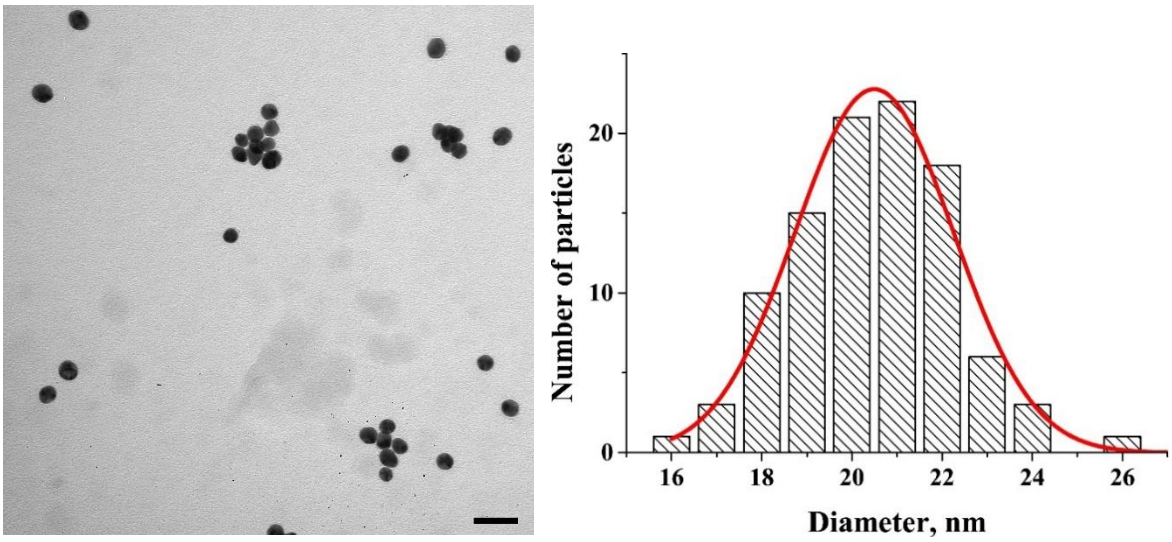

(a) (b)

Figure S1. Characteristics of Au NPs. (**a**) Microphotographs of Au NPs obtained by TEM (the bare scale is equal to 50 nm); (**b**) Distribution of Au NPs by diameters.

S2. EDS spectra of nanoparticles

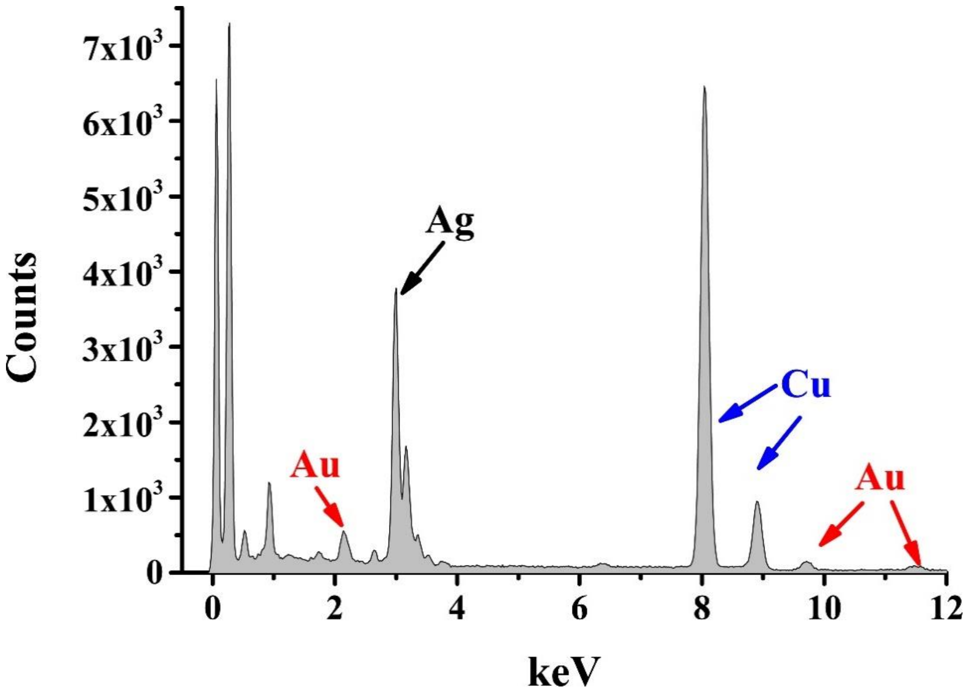

(a)

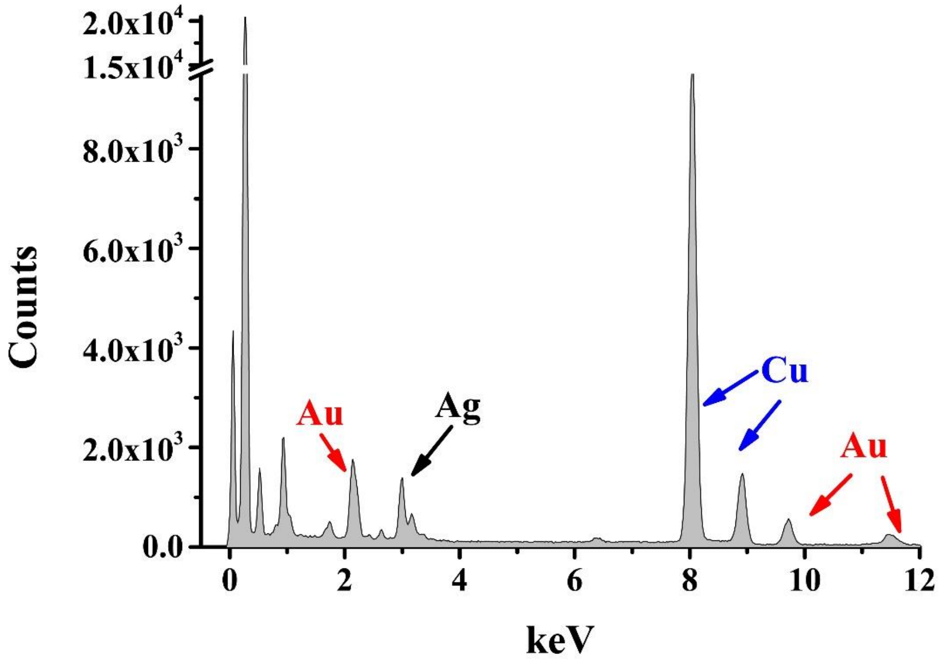

(b)

Figure S2. EDS spectra of nanoparticles. (**a**) Au@Ag nanoparticles (after silver enhancement); (**b**) Au@Ag-Au nanoparticles (after galvanic assisted Au deposition).

S3. Transmission electron microscopy of Au@Ag-Au nanoparticles

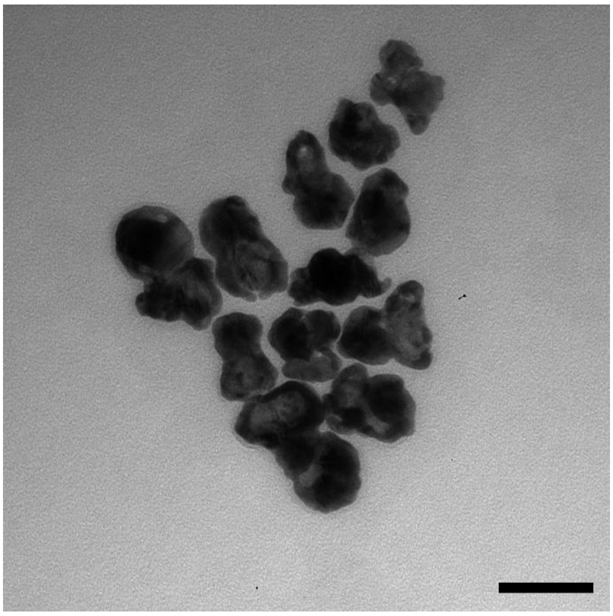

(a)

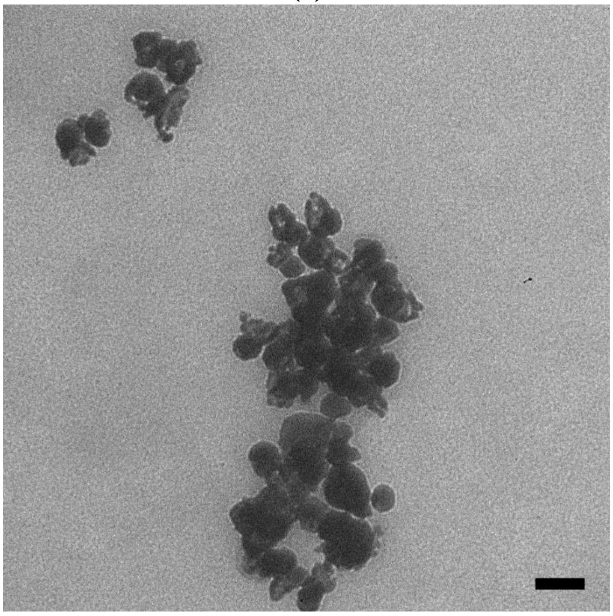

(b)

Figure S3. Microphotographs of Au@Ag-Au nanoparticles. The bare scale is equal to 50 nm.

S4. Characterization of the composition of Au@Ag nanoparticles

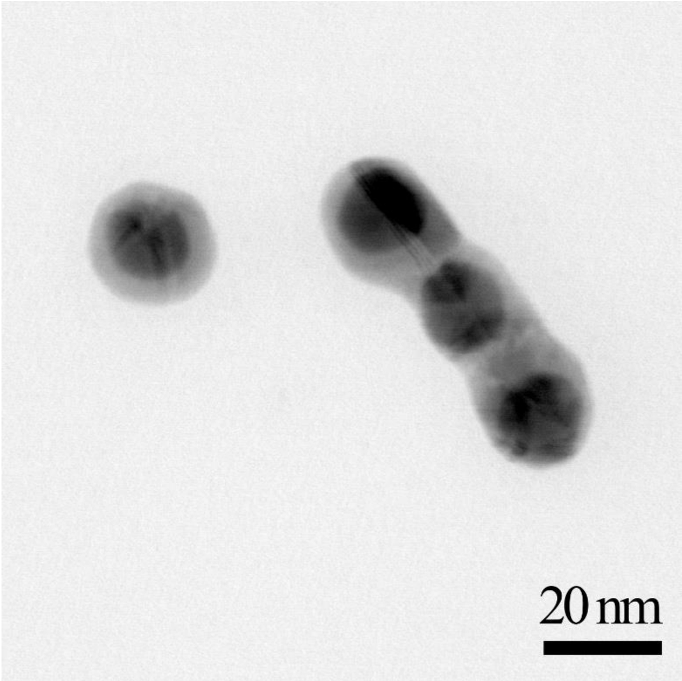

(a)

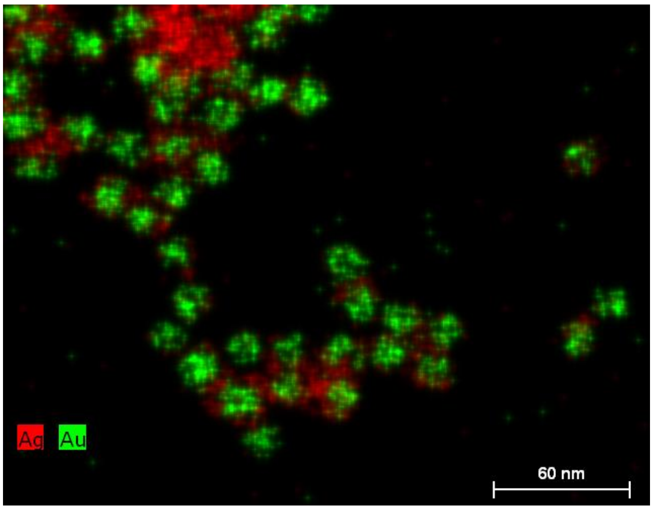

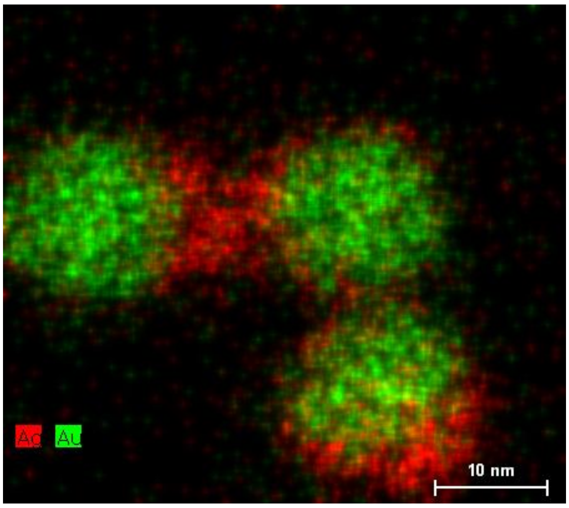

(b) (c)

Figure S4. Microphotographs of Au@Ag NPs. (**a**) TEM microphotograph of nanoparticles; (**b, c**) energy-dispersive spectroscopy mapping of chemical elements in Au@Ag nanoparticles. Ag atoms are shown in red; Au atoms are shown in green.

S5. Characterization of the composition of Au@Ag-Au nanoparticles

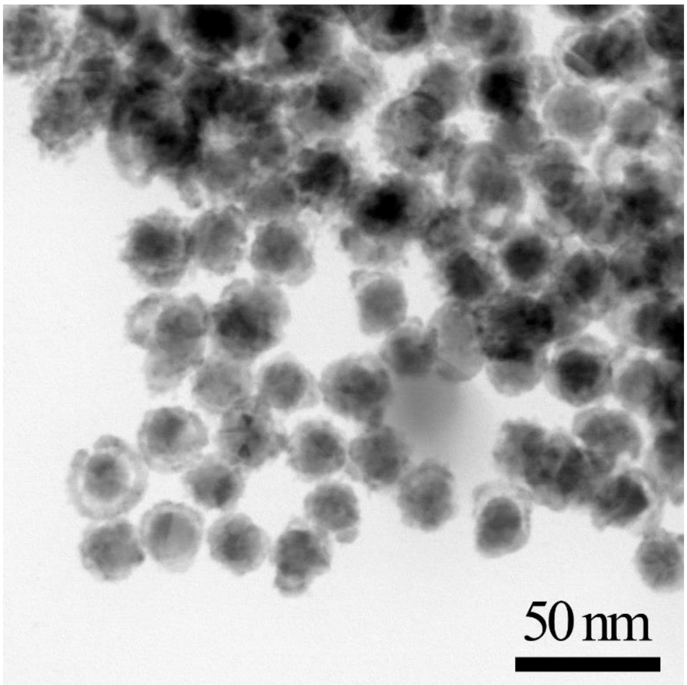

(a)

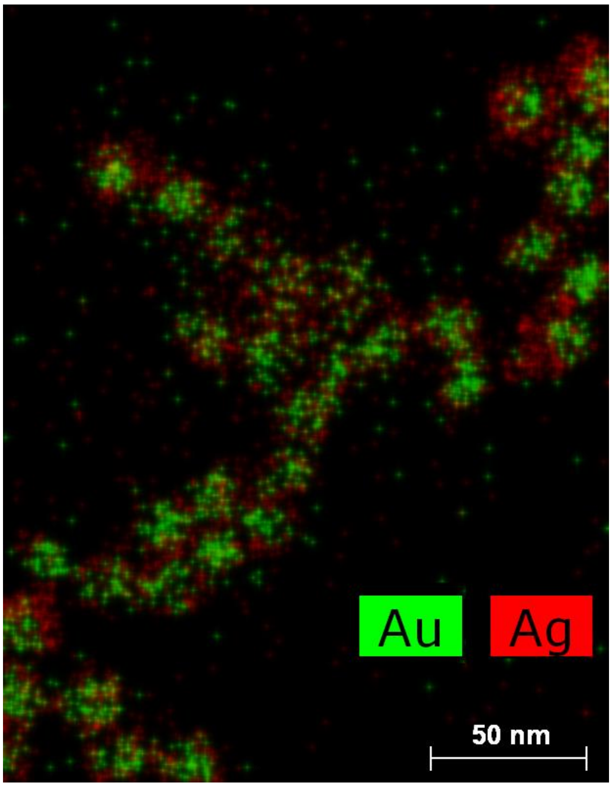

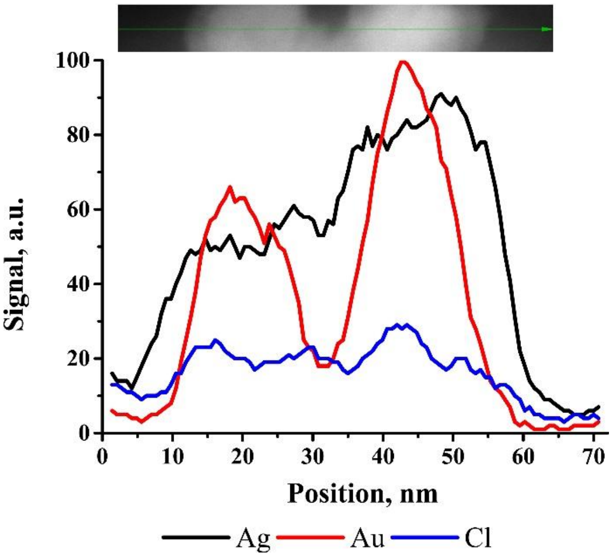

(b) (c)

Figure S5. Microphotographs of Au@Ag-Au NPs. (**a**) TEM microphotograph of nanoparticles; (**b**) energy-dispersive spectroscopy mapping of chemical elements in Au@Ag-Au nanoparticles; (**c**) the line profiles demonstrating distribution of chemical elements. The mapping was performed as shown by the green arrow in the TEM image

S6. Evaluation of the Au and Ag precursor consumption

The prices for precursors were taken from the official website (https://www.sigmaaldrich.com/) on June 2021. The consumption of the precursors were calculated on 1, 000, 000 tests for all four formats. The price of the precursors was recalculated for the single test (Table S1).

The price of the precursors for the single test is equal to 0.00042 $ (LFIA with Au NPs), 0,00047 $ (LFIA with silver enhancement), 0.00383 $ (LFIA with galvanic assisted Au deposition), 0.00218 $ (LFIA with gold enhancement)

Table S1. Consumption of Au and Ag precursors for the developed LFIA formats

Stage	LFIA with Au NPs	LFIA with silver enhancement	LFIA with galvanic assisted Au deposition	LFIA with gold enhancement
Required amount of the conjugate, A_{520}=4 L (1)	4.5	4.5	4.5	4.5
Required amount of Au NPs for the conjugates synthesis	18	18	18	18
Required amount of $H[AuCl_4]$, g (2)	2	2	2	2
Price of the required amount of $H[AuCl_4]$, (cat # 59790), $	416	416	416	416
Required volumes of enhancement solutions, L	No enhancement	5 L of 2% hydroquinone<br><br>5 L of 0.2% silver nitrate (3)	5 L of 2% hydroquinone<br><br>5 L of 0.2% silver nitrate (3)<br><br>10 L of 5 mM $H[AuCl_4]$, (4)	5 L of 5 mM $H[AuCl_4]$, 4<br><br>5 L of 1 M H_2O_2 (5)
The total price of the precursors for 1, 000, 000 tests, $	416	473 (6)	3833 (7)	2184 (8)

(1) 1, 000, 000 tests correspond to 3, 000, 000 mm of a glass-fiber membrane (3 mm width test strip) and require 4, 500, 000 µL (4.5 L) of Au NP conjugate

(2) The used method of Au NPs synthesis required 40 µL of 25% $H[AuCl_4]$, for the synthesis of 0.1 L of Au NPs. Thus, 7200 µL of 25 % $H[AuCl_4]$, is required for 18 L. Taken into account possible losses, 2g of $H[AuCl_4]$, (equal to 8000 µL of 25 % $H[AuCl_4]$,) is required.

(3) The mixture of 2% hydroquinone (5 µL) and 0.2 % silver nitrate (5 µL) was added to the test strip. Thus, 1, 000, 000 tests require 5,000,000 µL of the hydroquinone and silver nitrate solutions.

(4) The solution (10 µL) containing 5 mM $H[AuCl_4]$, was added to the test strip. Thus, 1, 000, 000 tests require 10 L of 5 mM $H[AuCl_4]$.

(5) The mixture of 5 mM $H[AuCl_4]$, (5 µL) and 1 M H_2O_2 (5 µL) was added to the test strip. Thus, 1, 000, 000 tests require 5, 000, 000 µL of 5 mM $H[AuCl_4]$, and 1 M H_2O_2.

(6) 100 g of hydroquinone (#H9003, 100g, the price is equal to 27.2 $) and 10 g of silver nitrate (#209139, 25g, the price is equal to 141 $. Thus, the required amount cost about 57 $).

(7) Dissolution of 20 g of $H[AuCl_4]$, in 10 L (5.9 mM) require 4 packages (#50790, 5g, 840 $). Thus, the required amount costs 3360 $).

(8) Dissolution of 10 g of $H[AuCl_4]$, in 5 L (5.9 mM) require 2 packages (#50790, 5g, 840 $. Thus, the required amount costs 1680 $). Dilution of 500 mL of 30 % H_2O_2 (1072090500, 500 mL, 88.1 $) to 5 L will provide 1 M H_2O_2.

S7. Calibration of LFIA with galvanic assisted Au deposition

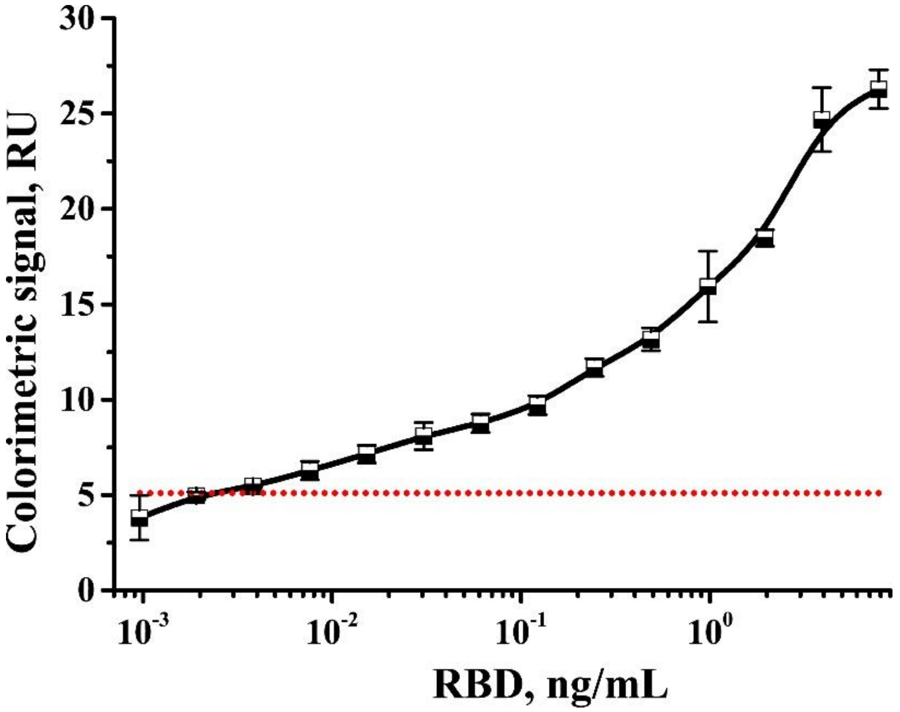

Figure S6. Calibration curve for LFIA after galvanic assisted Au deposition at low RBD concentrations. The red dotted line corresponds to the colorimetric signal equal to the mean background + 3SD.

S8. Calibration of LFIA with gold enhancement

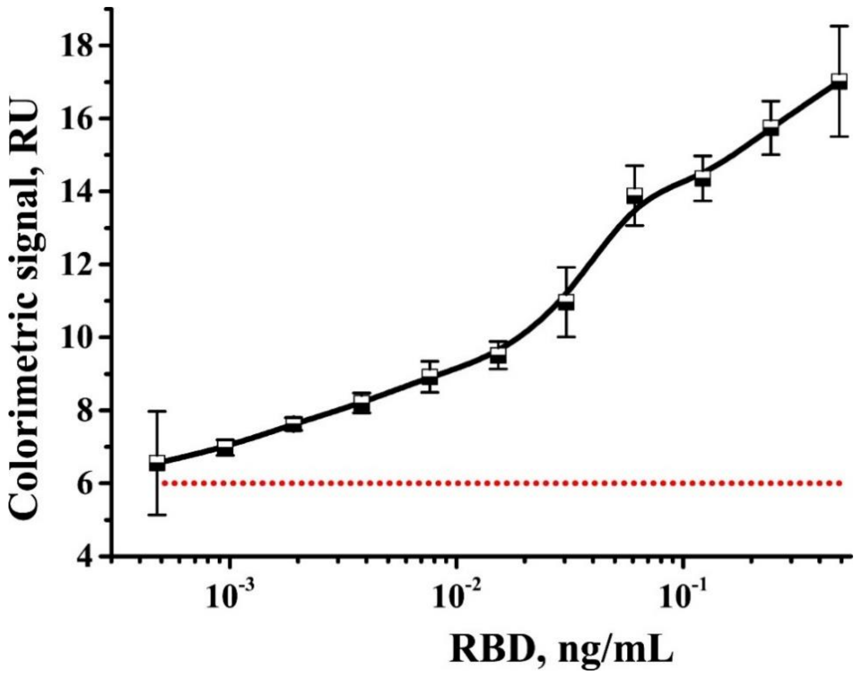

Figure S7. Calibration curve for LFIA after gold enhancement at low RBD concentrations. The red dotted line corresponds to the colorimetric signal equal to the mean background + 3SD.

S9. Validation of LFIA with Au NPs and gold enhancement

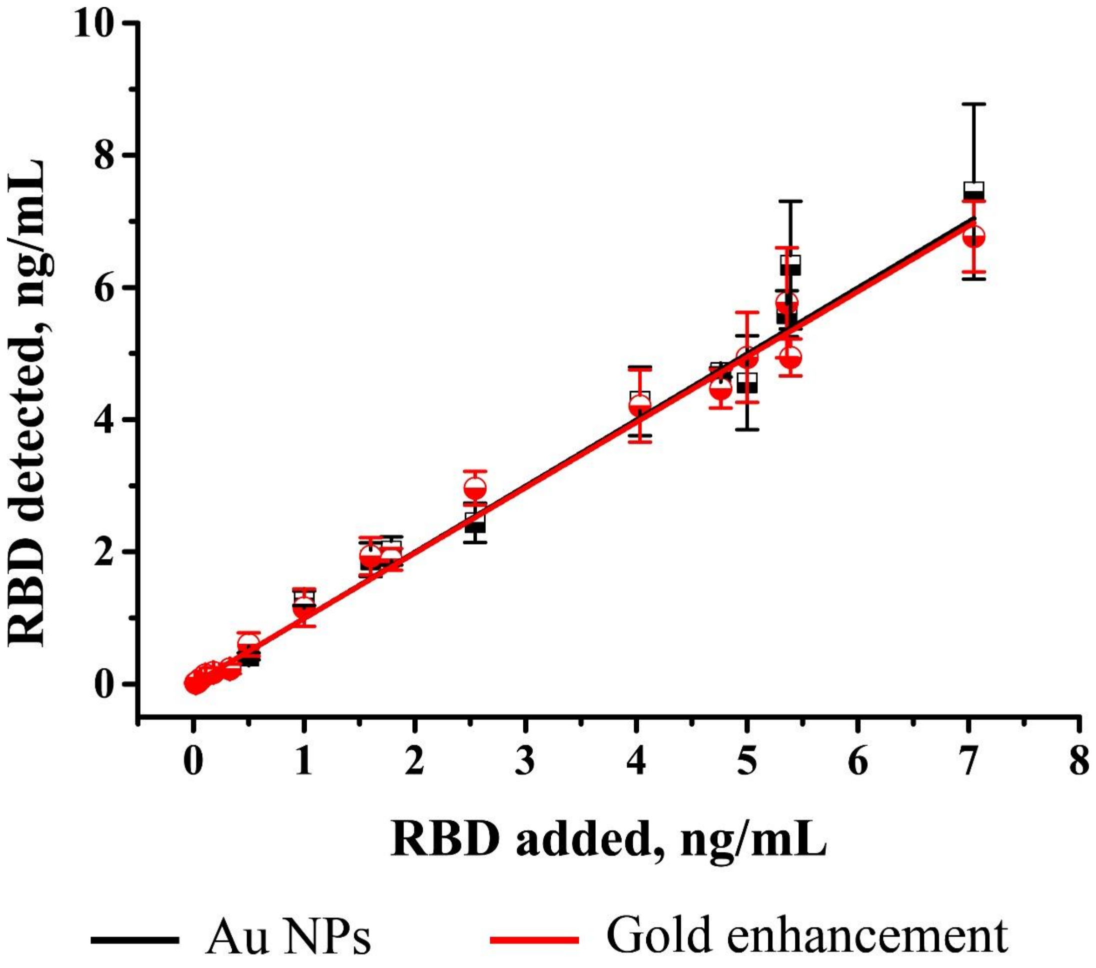

Figure S8. Correlation between added and detected concentrations of RBD for LFIA with Au NPs and gold-enhanced LFIA. Black and red lines correspond to the linear fitting (R^2 = 0.98)
